# An automated image-based dietary assessment application: a pilot study

**DOI:** 10.1017/jns.2025.10045

**Published:** 2025-11-04

**Authors:** Lachlan Lee, Rhiane Bishop, James Stanley, He Rourou Whai Painga Consortium, Jeremy David Krebs, Rosemary Hall, Richard Gearry, Troy L. Merry, Andrea Braakhuis, Anna Worthington, Fiona E Lithander, Meika Foster, Anna Rolleston, Amber Parry-Strong, Cecilia Ross, Mark Weatherall, Denise Conroy, Cheryl Davies

**Affiliations:** 1 Department of Medicine, University of Otago Wellington, Wellington, New Zealand; 2 Centre for Endocrine, Diabetes, and Obesity Research, Wellington, New Zealand; 3 Biostatistics Group, University of Otago Wellington, Wellington, New Zealand

**Keywords:** Diet, Dietary assessment, Mobile applications, Nutrition, Energy intake, EIapp, Energy Intake estimated by the App, EIrecall, Energy Intake estimated by the 24-hour recalls, VO2, Oxygen consumption, EE, Total energy expenditure, AEE, Activity related energy expenditure, REE, Resting energy expenditure, DIT, Diet-induced thermogenesis, ENMO, Euclidean Norm Minus One

## Abstract

Accurate assessment of an individual’s diet is vital to study the effect of diet on health. Image-based methods, which use images as input, may improve the reliability of dietary assessment. We developed an iOS application that uses computer vision to identify food from images. This study aimed to assess the accuracy of energy intake (EI_app_) estimates from the application by comparing them to estimated energy expenditure (EE) and to the EI estimates from a validated dietary assessment tool, the 24-h recall (EI_recall_). Participants were recruited from a randomised controlled trial called He Rourou Whai Painga. Participants recorded all intake over 7 d using the application, which provided a mean daily EI; this was compared to the EI estimated by two 24-h recalls. The EI from the application and the recalls were compared to EE, estimated using indirect calorimetry and wrist-worn accelerometry. EI estimates from the application and the 24-h recalls were lower than EE, with a mean bias of -1814 kJ (95% CI -3012 to -615, *p* = 0.005) and -1715 kJ (95% CI -3237 to -193, *p* = 0.029), respectively. The mean bias between EI from the application and the 24-h recall was 783 kJ (95% CI -875 to 2441, *p* = 0.33). This suggests that the EI estimates from the application are comparable to the 24-h recall method, a validated and widely used tool in nutritional research.

## Introduction

The prevalence and health impact of diabetes, obesity, and CVD are increasing in New Zealand and worldwide.^(1,2)^ The relationship between diet and cardiometabolic disease is well established. However, the tools used to assess diet are often inaccurate. Though dietary assessment produces rich data, the accuracy of dietary assessment is most often gauged using energy intake (EI), where all reporting methods are known to underestimate EI. Under-reporting of EI limits the assessment of individual or population dietary patterns.^(3)^ Dietary intake is traditionally assessed using written, text-based, self-recorded food records or assisted recall methods. These are prone to recall bias and have a high level of participant burden.^(4)^ Dietary assessment tools can be improved using the many advances in computer hardware, software, and artificial intelligence, and a variety of methods have been developed for text or image-based dietary assessment.^(5)^ Text-based methods use text search to identify the food or beverage being consumed, similar to using a search engine, whereas image-based methods use an image instead of text.^(4,6,7)^ Image-based input can be partly automated by suggesting food items from a database that are visually similar to the image provided.^(6,7)^ Image-based and text-based methods are complementary; capturing images can be quicker and simpler than text-based input and is generally preferred by participants, but unlike text-based, input images cannot be recorded after consumption^(8)^.

Our research group aims to develop an open-source dietary assessment tool for clinical and research use. Ensuring the tool is well-designed, acceptable to participants living in Aotearoa New Zealand (AoNZ), and generates accurate dietary records requires iteration through testing in free-living individuals. We have developed the first version of this tool: an iOS phone application, termed here the ‘App’, that offers both text-based and automated image-based input.

The first objective of this study was to compare EI estimates from the App (EI_app_) to estimated energy expenditure (EE). The second objectives were (a) to compare EI estimated by the App to that estimated by a validated 24-h recall dietary assessment tool and (b) to compare EI estimated by the 24-h recall to EE. The third objective was to explore the user experience of the App to identify strengths, limitations, and areas for improvement for future development.

## Methods

### Ethics

This study was conducted according to the guidelines laid down in the Declaration of Helsinki, and all procedures involving human subjects/patients were approved by the Health and Disability Ethics Committee as a sub-study to the He Rourou Whai Painga trial (2022 FULL 12045). Written informed consent was obtained from all subjects/patients.

### Study design

**Fig. 1.**
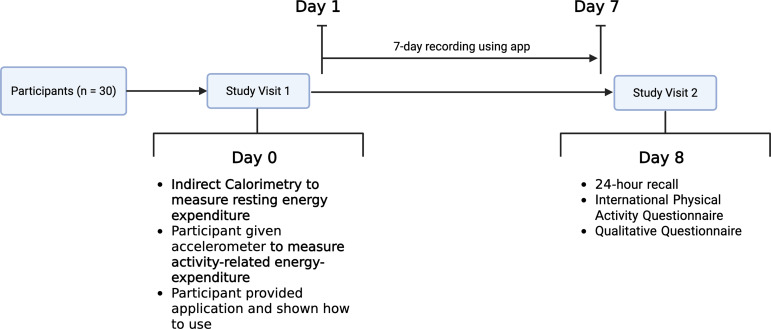
Overview of study design.

This was a single-arm cross-sectional study comparing EI estimates from seven consecutive days of dietary assessment from the App, with estimations of EE (Fig. 1).

### Recruitment

This study recruited Wellington-based participants in the He Rourou Whai Painga randomised controlled trial aged 18 years and over as a pre-identified sub-study within the larger trial.^(9)^ The He Rourou Whai Painga trial aimed to test whether an intervention including a Mediterranean dietary pattern incorporating high-quality New Zealand foods and behaviour change science could improve the metabolic health of participants and their households.

In total, there were 124 participants in the Wellington region, and the current study aimed to recruit 30 of those 124 participants. The protocol paper for the He Rourou Whai Painga trial is detailed in Lithander *et al.* and will be briefly detailed here.^(10)^ The He Rourou Whai Painga trial included index participants and their households. Inclusion criteria for index participants were as follows: aged 18–70 years and metabolic syndrome severity score >0.35. Inclusion criteria for the household participants were consent or assent to consume the intervention food provided through the He Rourou Whai Painga trial. Exclusion criteria for index and household participants were as follows: previous bariatric surgery, pre-existing type 1 diabetes mellitus or type 2 diabetes mellitus in the index participant, total cholesterol ≥8 mmol/L, severe renal impairment (estimated glomerular filtration rate of <30 mL/min/1.72m^2^), current pregnancy or intention to conceive during the study period, active weight gain or loss of >5 kg in the prior 3 months, gastrointestinal disorders that affect the digestion or absorption of nutrients (e.g., ulcerative colitis, Crohn’s disease, coeliac disease), anaphylaxis to food items for either index participant or their household, use of medications that modify blood sugar levels, anticipated use of oral or injected steroids, does not agree to refrain from donating blood due to its effect on HbA1c, or any other condition or situation which in the view of investigators would affect the compliance or safety of the individual taking part.

In addition to the inclusion and exclusion criteria for He Rourou Whai Painga, the inclusion criteria for this study were as follows: (1) aged 18 years or over, (2) access to a camera-capable iPhone operating system (iOS) device running iOS 16 or later, (3) access to the internet, and (4) able to speak and read the English language. Inclusion criteria 2 and 4 were required as the current version of the App is iOS-only and only available in English. There were no specific additional exclusion criteria.

An important element of research in Aotearoa New Zealand is to include Māori, the indigenous people of Aotearoa, as research participants. This was of particular importance in this study as informed design based on Māori perspectives is a wider goal for continuing development of the App. This was a priority in the He Rourou Whai Painga trial, and was enabled through the close relationship between the Wellington-based research site and the Tū Kotahu Māori Asthma and Research Trust at Kōkiri Marae in Lower Hutt, a community-based traditional Māori meeting place.

### Outcomes

The primary objective was to assess the agreement of EI recorded using the image-based dietary assessment App and estimated EE. Secondary objectives included the agreement of estimated EE compared to the EI recorded using two 24-h recalls (Intake24, University of Cambridge), the agreement of EI recorded using the App compared to the EI recorded using the 24-h recalls, and the user experience of the App.

### Energy Intake

Participants recorded their EI using the new automated image-based dietary assessment App over a 7-d period between visit 1 and visit 2. At visit 2, participants completed a 24-h recall, the New Zealand-adapted Intake24 (Newcastle University), that examined the 24-h period of day 7. The EI on day 7 was therefore estimated by both the App and the 24-h recall. The App was designed in collaboration with students from the Master’s of User Experience Design programme at Te Herenga Waka Victoria University of Wellington (New Zealand) and developed with software engineers at Deviark LLC (Lviv, Ukraine). The design process involved a literature review of current image-based dietary assessment tools and iterative designs based on user testing conducted by the Master’s of User Experience Design programme. The App offers three key features: (1) a dashboard that displays daily energy and macronutrient consumption, (2) text-based input through a searchable database, and (3) automated image recognition using Passio Nutrition AI software (Passio Inc., California, USA). The food recognition software uses machine learning techniques; descriptions of similar techniques can be found elsewhere.^(6,7)^ The image-based input was activated through a button in the App; the participant could then see the camera view, similar to the display shown prior to capturing an image on a smartphone camera. The Passio Nutrition AI software attempts to identify any potential food items included in this view and suggests matching database item(s) in a pop-up menu. Participants select the matching item to record intake. Text-based input involves typing the appropriate words into a search bar and selecting the matching database item(s). No images or videos of the food item are recorded or stored. Participants used the image-based and text-based input methods to record individual food items, with energy and macronutrient data associated with each food item within the Passio database. The proprietary Passio database is developed and maintained by Passio Inc. (California, USA) and is used in popular dietary assessment applications such as MyFitnessPal (MyFitnessPal Inc.). Participants were able to edit the serving size of each item as required using a slider, similar to a volume slider. The App stored data in a secure cloud-based database hosted by Firestore (Alphabet Inc., USA) in a username- and password-secured account. The database was in a non-SQL structure, which was exported to a comma-separated values Format file for subsequent analysis. The main functionalities of the App and a brief guide to its use can be found in Appendix (A).

Participants were asked to continuously record all their dietary intake from Visit 1 (day 0) to Visit 2, with at least seven consecutive days between each visit. Only data recorded from days 1–7 were used to assess agreement with estimated EE, as participants were required to fast for measurement of resting VO_2_ via indirect calorimetry on day 0, and both days 0 and 8 were incomplete recordings due to the study visits. EI estimations from the App (EI_app_) and the 24-h recall (EI_recall_) were assessed for agreement. The mean EI estimations from the App were collected over 7 d, the mean of which was compared to the EI estimations from the 24-h recalls; see the section on 24-h recalls for further details. The 24-h recall, even when administered multiple times, can be affected by within-subject variation as it assesses fewer number of days compared to food records such as the App. In practice, the EI recorded using 24-h recalls is assumed to represent EI over time, ideally by administering the recall two or more times.^(11–14)^ Therefore, assessing for agreement between the two methods is practical. Energy intake results are reported by sex to represent the recognised differences in EI by sex.^(15)^

#### 24-h Recall

At Visit 2, participants were asked to complete a 24-h recall hosted on Intake24 (Newcastle University, United Kingdom). As part of the wider He Rourou Whai Painga study, participants completed 24-h recalls at multiple timepoints. The 24-h recall completed at sub-study Visit 2 and a 24-h recall within 30 d of the sub-study recall were included in the analysis. Intake24 is a self-reported computerised 24-h recall; participants initially use free-text to record their intake and then match this input to items in the database, assisted by images of food items and portion sizes.^(16)^ The Intake24 database used in this study has been adapted for use in New Zealand by Follong *et al*. in response to the recommendations provided by Mackay *et al.*. ^(11,17)^ These adaptations incorporated cultural dishes specific to the indigenous Māori population, adding new portion size estimation aids, and customising the user interface of Intake24. Participants were asked to record all dietary intake from the previous 24-h day, midnight to midnight. Participants were supervised by either LL or RB to ensure that they did not use the App to assist with the recall during the sub-study Visit 2 recall. The mean EI from the two 24-h recalls was compared to the mean EI over the 7 d of recording with the App and the mean EE over the 7 d. The recalls were excluded from analysis if they were not completed within 30 d of each other. Increasing the number of recalls would reduce the effect of day-to-day variation in EI. However, this must be balanced with participant burden, particularly during intensive studies and sub-studies. The use of two 24-h recalls, a validated method of assessing EI at the group-level, therefore balances the effect of day-to-day variation with participant burden.^(18–20)^

### Energy Expenditure

Energy expenditure comprises resting energy expenditure (REE), activity-related EE, and diet-induced thermogenesis (DIT). Total EE (kJ/d) was calculated for days 1–7 by adapting the method described by Hibbing *et al.*. ^(21,22)^ This adapted method involves the use of indirect calorimetry to measure resting oxygen consumption (VO_2_) and wrist-worn accelerometers to estimate total EE.

#### Resting oxygen consumption

Resting oxygen consumption was measured by indirect calorimetry using the PromethION High-Definition Room Calorimetry System (Sable Systems International, USA) according to the protocol detailed by Corley.^(23)^ The preconditions for the measurement of REE were used, rather than basal metabolic rate (BMR), as the preconditions for BMR measurement were not feasible within this study. Participants attended after an overnight fast of at least 8 h, except for free intake of water, and were asked to refrain from moderate or vigorous physical activity the day of the measurement. Each participant remained in the indirect calorimeter for 30–60 min watching a documentary on an iPad (Apple Inc., USA) in a reclined position, in a thermoneutral room. Data were collected once a steady state was reached with equilibration of room gases. Data were processed in CaloScreen software (Sable Systems International, USA).

#### Total Energy Expenditure

Total EE was estimated using the resting VO_2_ measurement from indirect calorimetry and a tri-axial accelerometer, the Actigraph GT9X (ActiCorp Ltd., USA). Participants were instructed to wear the device on their non-dominant hand and only to remove it for swimming and bathing. Acceleration data from the ActiGraph GT9X Link were exported as raw.gt3x files, which were processed using GGIR (v3.2.0) by combining the three axes of acceleration data (in milli-gravitational units) into a single variable, the Euclidean Norm Minus One, in one-second epochs. Accelerometer data was converted from.gt3x to.agd via ActiLife (v6.13.6) and screened for non-wear in 1-min epochs using the method proposed by Choi *et al.* ^(24)^ and for sleep using the method proposed by Tracy *et al*. ^(25)^ Valid days were defined as having ≥10 h of wear-time, with invalid days excluded from analysis. For minutes marked as either non-wear or sleeping minutes, resting rVO_2_ was imputed. Negative values were rounded up to 0. The per-second ENMOs were converted into VO_2_ using the non-linear Hildebrand equation.^(26)^ The method described by Hibbing was adapted here by setting the minimum VO_2_ for each participant as the resting VO_2_ measured by indirect calorimetry, whereas Hibbing *et al.* used a uniform floor of 3.0 mL/kg/min for all participants. A ceiling of 70 mL/kg/min was applied. VO_2_ was converted to kilocalories assuming a respiratory quotient of 0.85, or 4.862 kcal/L, and subsequently converted to kilojoules using a ratio of 1 kcal:4.184 kJ.^(27)^ The per-minute EE is summed within each day, and a mean is calculated across all days recorded.

### Anthropometry

Anthropometric measures include height (cm), weight (kg), and body composition metrics such as fat-free mass (kg). Height was measured using a calibrated wall-mounted stadiometer. Weight and body composition metrics were measured using a bio-electrical impedance scale (TBF-400, Tanita Corporation, Arlington Heights, IL).

### User Experience Questionnaire

In Visit 2, participants were asked to complete an eleven-item questionnaire exploring the user experience of the App. The questionnaires were adapted from the System Usability Scale and the Mobile App Rating Scale by emphasising simple language and promoting open-ended responses, and are intended to identify specific areas for iterative improvement in this App.^(28,29)^ The questionnaire featured a combination of free-text responses and visual analogue scales. The visual analogue scales were unmarked lines with text descriptions describing the extreme ends of responses, for example, ‘very likely’ or ‘not likely at all’. The full questionnaire can be found in Appendix (B) and explores participants’ likes, dislikes, surprises, frustrations, missing features, subjective experience of image-based input accuracy, and overall experience with the App.

#### Statistical Analysis

Analysis of agreement used a Bland–Altman approach.^(30)^ Visual inspection of Q-Q plots and a histogram of the model residuals was checked for homogeneity of variance or any obvious deviations from normality. Analysis also included calculating the mean, SD, and CI and performing paired t-tests comparing EI_app_ versus EE, EI_recall_ versus EE, and EI_app_ versus EI_recall_. All inferential analyses report 95% CI and use an alpha of 0.05 for hypothesis tests. A sample size of 30 allows for sufficient degrees of freedom (greater than 20) to estimate a variance with reasonable precision. The sample size for the study was set based on recruitment feasibility in the context of the larger trial and the expected meeting of eligibility criteria. No formal sample size calculation was conducted. Statistical analysis was performed using R 4.2 (R Foundation, Vienna, Austria), and statistical software was used with the blandr and ggplot2 packages.^(31,32)^

## Results

### Participants

**Fig. 2.**
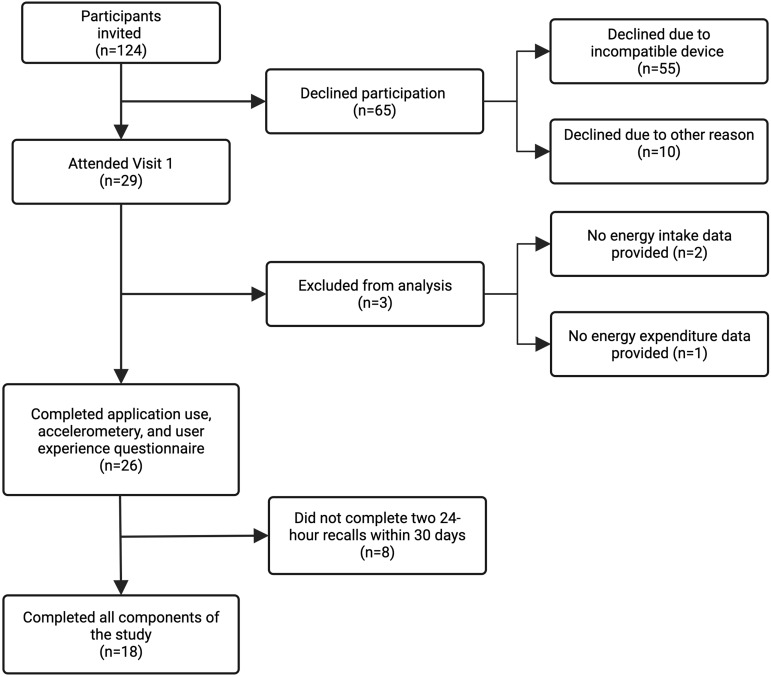
Diagram of recruitment and participation flow through the study.

A total of twenty-nine participants were recruited. The recruitment process is shown in Fig. 2. All 124 potential participants were contacted by email, and 94 responded (76%). Of these, sixty-five (68%) declined to participate. Of those who declined participation, fifty-five (85%) owned an Android device, which was incompatible with the App, and ten (15%) did not wish to participate. There were twenty-nine participants; 23% of the available participant pool and 30% of respondents, who attended Visit 1. Nineteen participants completed all components of the study. Two participants did not provide any data for EI_app_, and one participant did not provide any EE data via the accelerometer. The participant who did not provide EI_app_ and the participant who did not provide EE data were not included in the data analysis. One participant was unable to complete anthropometry assessments at Visit 2 due to COVID-19 infection but was included in the analysis. Eight participants did not complete two 24-h recalls within 30 d and were not included in the analysis involving the 24-h recalls but were included in other analyses.

Participant baseline characteristics for the twenty-nine participants are shown in Table 1.

**Table 1.** Participant baseline characteristics

Continuous variables	Mean (SD^a^)
**Age (years)**	47 (12)
**Weight (kg)**	98.9 (25.7)
Male	110 (32.9)
Female	95.8 (22.6)
**Height (cm)**	167.5 (9.7)
Male	177.8 (10.1)
Female	164.5 (6.9)
**BMI** ^b^ **(kg/m** ^2^ **)**	35.2 (8.1)
Male	34.6 (8.9)
Female	35.4 (7.9)
**Fat mass (%)**	37.2 (10.4)
Male	27.6 (8.1)
Female	40.1 (8.9)
**Categorical variables**	**n/27 (%)**
**Sex**	
Male	8 (30%)
Female	19 (70%)
**Ethnicity**	
New Zealand European	15 (56%)
Māori	11 (41%)
Pacific	1 (4%)
Other	3 (11%)

a Standard deviation

b body mass index

### Energy Intake

The mean and SD of the ratios between EI_app_ and EE, EI_app_ and EI_recall_, and EI_recall_ and EE are reported in Table 2. The mean and SD of the absolute values of EI_app_ and EI_recall_ can be found in Appendix C.

**Table 2.** Mean and SD of resting VO_2_^a^, estimated EE^b^, the ratio of EI_app_^c^ and estimated EE, the ratio of EI_recall_^d^ and estimated EE, and the ratio of EI_app_ and EI_recall_

Sex	Mean resting VO_2_ (mL/kg/min) (SD, n)	Mean estimated EE (SD, n)	Mean EI_app_:EE (SD, n)	Mean EI_recall_:EE (SD, n)	Mean EI_app_:EI_recall_ (SD, n)
Male	2.79 (0.50, 8)	10519 (3522, 8)	0.81 (0.25, 8)	0.78 (0.28, 5)	1.21 (0.19, 5)
Female	2.62 (0.38, 19)	8349 (1873, 18)	0.87 (0.44, 18)	0.88 (0.37, 13)	1.23 (0.60, 13)
Total	2.67 (0.42, 27)	9017 (2628, 26)	0.85 (0.39, 26)	0.85 (0.34, 18)	1.22 (0.52, 18)

a VO_2_: Oxygen consumption

b EE: mean of energy expenditure estimated by indirect calorimetry and accelerometry across the observation period

c EI_app_: mean of daily energy intake across the observation period

d EI_recall_: mean of energy intake between two 24-h recalls completed within 30 d

#### *Comparison of EI*_*app*_ *, EE, and EI*_*recall*_

**Fig. 3.**
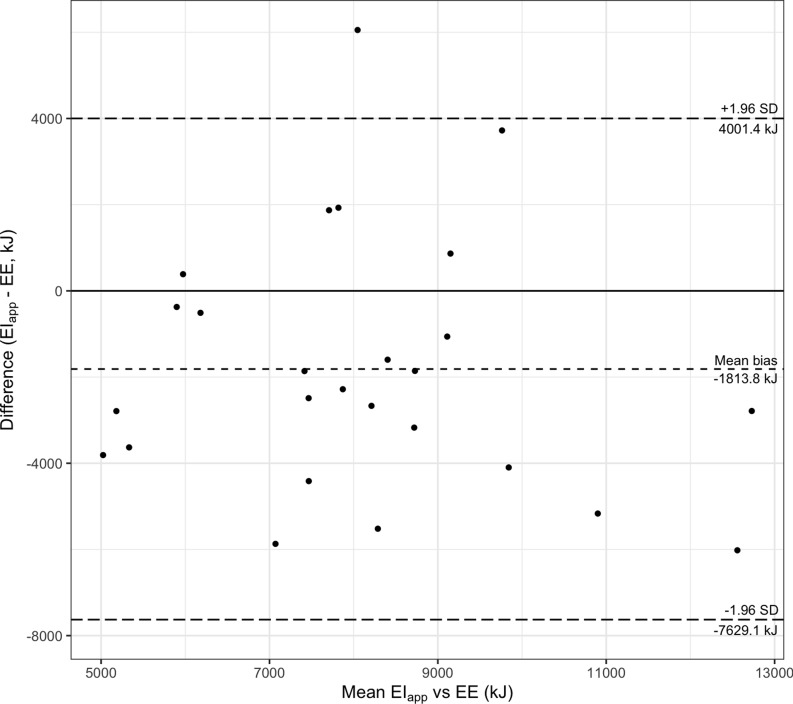
Bland–Altman plot of EI_app_^a^ (kJ/d) and EE^b^ (kJ/d). ^a^ EI_app_: mean of daily energy intake across the observation period. ^b^ EE: mean of energy expenditure estimated by indirect calorimetry and accelerometry across the observation period.

Summaries of the ratio of EI estimated by the App and estimated EE are shown in Table 3. The following comparisons were made to assess agreement: EI_app_ and EE, EI_recall_ and EE, EI_app_ and EI_recall_. The Bland–Altman plot for this comparison is shown in Fig. 3. The Bland–Altman plot for the comparison between EI_recall_ and EE and EI_app_ and EI_recall_ is found in Appendix (C). The Bland–Altman analyses for the comparison between EI_app_ and EE, as well as the comparison between EI_recall_ and EE, showed that EI_app_ and EI_recall_ were lower than EE, and the comparison between EI_app_ and EI_recall_ showed a relatively smaller mean bias.

**Table 3.** Bland–Altman analyses of EI_app_^a^ (kJ/d) versus estimated EE^b^ (kJ/d), EI_recall_^c^ (kJ/d) versus EE, and EI_app_ versus EI_recall_

Comparison	Estimated mean bias (95% CI, p-value)	Limits of agreement
**EI**_**app**_ **vs EE (n = 26)**	-1814 kJ (-3012 to -615, p = 0.005)	-7629 to 4001
**EI**_**app**_ **vs EI**_**recall**_ **(n = 18)**	783 (-875 to 2441, p = 0.33)	-5751 to 7317
**EI**_**recall**_ **vs EE (n = 18)**	-1715 (-3237 to -193, 0.029)	-7714 to 4285

a EI_app_: mean of daily energy intake across the observation period

b EE: mean of energy expenditure estimated by indirect calorimetry and accelerometery across the observation period

c EI_recall_: mean of energy intake from two 24-hour recalls

### Energy Expenditure

Mean EE estimated by indirect calorimetry and accelerometry is reported in Table 2. The mean resting VO_2_ for female and male participants was 2.62 and 2.79 mL/kg/min, respectively. EE is reported by sex and by total population to represent the recognised differences in EE by sex.^(15)^

### User Experience Questionnaire

**Fig. 4.**
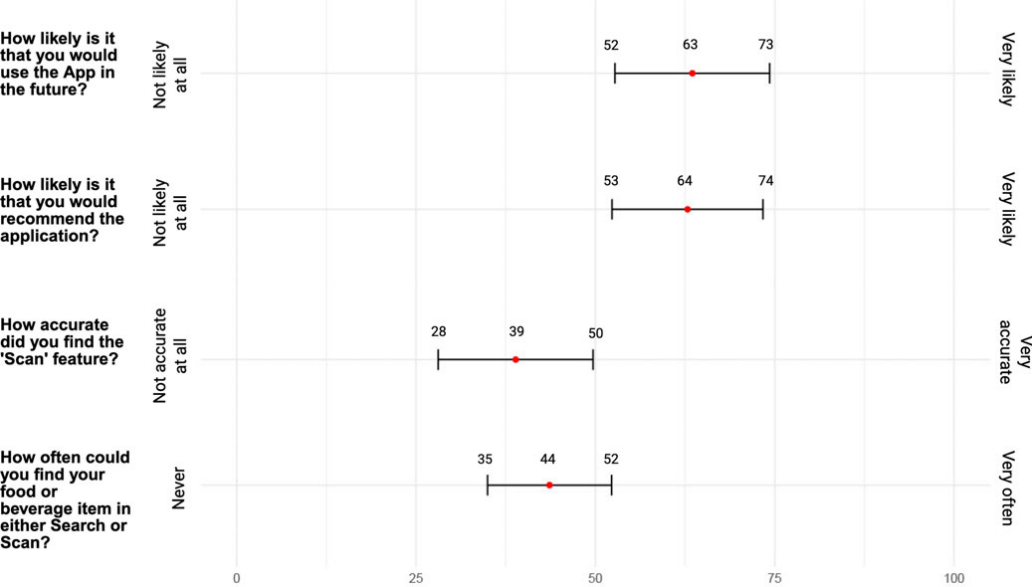
Mean and 95% CI of the User Experience Questionnaire Visual Analogue Scale responses.

A total of twenty-nine participants completed the User Experience Questionnaire. Fourteen of twenty-nine (48%) participants reported an overall positive experience with the App, ten participants (34%) reported a neutral or mixed overall experience, and four participants (14%) reported a negative overall experience with the App. When asked about the most liked part of the App, fifteen participants reported the automated image-based input, eight reported the user interface, and six participants cited the energy and macronutrient summaries provided by the App in real time. When asked about features they would like added to the App, participants suggested integration with a local food composition database, retrospective recording, reminder notifications, a daily review feature similar to a 24-h recall, flexibility in the units for editing serving size, physical activity tracking, and a ‘recipes’ feature, allowing users to combine food items in the database to make a novel food item, for example, combining ‘toast’ and ‘eggs’ to make ‘eggs on toast’. Twelve participants reported that their least favourite aspect of the App was when the database of foods did not have a food item that exactly matched their intake, while fourteen found this issue to be the most frustrating aspect of using the App. Ten participants reported that the absence of retrospective recording was the least enjoyable part of the App. Eight participants highlighted incorrect automated image recognition as a limitation of the App, and seven participants found this the most frustrating part of the App. Five participants reported difficulty in adjusting the size of portions in the App, with four citing this as the most frustrating aspect. App crashes and slow responsiveness were reported as the least enjoyable experience by six participants. Fifteen participants reported previous experience with a dietary assessment App; of these, nine participants had previously used MyFitnessPal. Figure 4 shows the mean and 95% CI of the visual analogue scale responses in the user experience questionnaire.

## Discussion

This prototype App showed comparable EI estimations to Intake24, a validated dietary assessment tool, though both methods likely under-reported compared to total EE.^(18,33,34)^ Participant feedback has identified several specific features that may improve the accuracy of the App’s EI estimates. A significant improvement to the App will be the inclusion of a food composition database more relevant to the New Zealand population, in keeping with validated and widely used tools such as Intake24.^(17)^

### Measuring energy intake

The EI estimates from the App and the 24-h recall were comparable, though the discrepancy between expenditure and intake suggests under-reporting. A similar disparity between reported EI and EE is consistently reported; Foster *et al*.^(18)^identified that EI is underestimated by 25% on average with Intake24. Lopes *et al.* ^(33)^ found EI was underestimated by 23% in men and 40% in women using an adapted US Department of Agriculture five-step multiple-pass method,^(35)^ and Lennox *et al.* ^(34)^ found mean underestimates of 25–35% in participants aged over 16 years, also using Intake24. In this study, the App and the 24-h recall both underestimated EI by 15%, approximating previous findings. In this study, the mean of 7 d of recorded EI using the App was assessed for agreement with the mean of 2 d of 24-h recalls administered within 30 d of each other. The larger number of days included for EI_app_ reduces within-subject error for EI_app_, thereby reducing the comparability between EI_app_ and EI_recall_. Assessing additional days using the 24-h recall would improve the comparability, but at the cost of participant burden. Additionally, the 24-h recall administered on day 7 of the study may have been affected by using the App on day 7. Participants may have improved recall of their intake as they have recorded the same day’s intake using the App; we attempted to address this by supervising the 24-h recall to ensure participants did not use the App to aid their recall. Conversely, participants may be prompted to complete the 24-h recall and revise the App’s dietary record for that day. In this study, we elected to use two 24-h recalls to improve the comparability of the methods without excessive participant burden or causing simultaneous use of the methods from affecting each other.

As the results are from a pilot study, with a focus on estimation of these different estimation approaches, no corrections were planned for multiple hypothesis testing. Applying a Bonferroni correction to set a new test-wise alpha level (0.05/3 = 0.0167) for significance for individual tests would return the same conclusions as noted here, these conclusions being no significant difference between EI_app_ and EI_recall_ and statistically significant differences between EI_app_ and EE and EI_recall_ and EE. Finally, while the accuracy of EI estimations is often used to infer the overall accuracy of a dietary record, there are more characteristics to a food item than total energy, including macro- and micronutrients. Accurate estimates of these characteristics may be important in different settings; the current App database contains micronutrient data and will be implemented in future versions. Therefore, future studies should include validation of macronutrient and micronutrient estimations as well, where feasible.

### Estimating energy expenditure

In this study, we adapted the method of estimating EE described by Hibbing *et al.* This method uses the non-linear Hildebrand equation to convert raw accelerometer signals into estimated VO_2_. Hibbing *et al.* process the estimated VO_2_ using filters that estimate if the accelerometer is not being worn, worn during sleep, as well as excluding days with less than 10 h of valid accelerometer wear-time, as defined by a separate filter. Because the non-linear Hildebrand equation can estimate VO_2_ below resting VO_2_, which is physiologically implausible, a minimum VO_2_ must be set. Hibbing *et al.* used a uniform 3.0 mL/kg/min for all participants; this uniform resting VO_2_ overestimates the resting VO_2_ in this study population and would therefore overestimate EE.^(22)^ Instead, the approach described in this study used the resting VO_2_ directly measured for each participant using indirect calorimetry. This may provide a more individualised estimation of EE. This approach does not add or subtract 10% of total EE to account for DIT, as the contribution of DIT to the non-linear Hildebrand equation is unclear. The non-linear Hildebrand equation was derived in participants who were fasted for only 2 h; therefore, the VO_2_ measurements may include some degree of DIT, as DIT can contribute to total EE for up to 10 h following consumption^(36)^. In this study, we elected not to add or subtract the standard 10% of total EE as a DIT correction, given the unclear degree of DIT factored into the equation.

### User experience of the app

The second main objective of this study was to understand the user experience to help guide further refinement and development of the App. The participants had mixed responses to the user experience of the App. The most frequently cited limitation and source of frustration was the food composition database within the App, which either did not feature the food item required by the participant or featured it under a different name. This is a significant limitation of this App and any dietary assessment tool that does not contain food and beverage items available locally to the user. The development and integration of a food composition database relevant and representative of the diet of the studied population is a requirement for accurate dietary assessment. However, even the most up-to-date and representative database will not contain all the food items that any individual user might require. This could be assisted by a ’Recipes’ feature, allowing users to create new food items from a food composition database by combining multiple food items together or by allowing users to manually input the energy and macronutrient content of their meals. However, this approach is dependent on users knowing the composition of the food they consume. A future additional feature could be the ability to scan the text of a recipe and integrate it into the database. Participants report that automated image-based input is a convenient and acceptable mode of dietary assessment, but its efficacy is limited by the associated database. Even within a particular country or region, diet composition can vary considerably, most notably varying by ethnicity. In a cross-sectional analysis of adults in Amsterdam, Yau *et al*.^(37)^ found significant variation between ethnic groups, while Huang *et al.* ^(38)^ similarly found nutrient intake variation between different ethnic groups in the United States. A food composition database that represents all diets across a range of ethnicities will be a requirement for accurate dietary assessment.

Participants in this study frequently forgot to record prior to consumption, which is a specific limitation of an image-based food record. This highlights the need for both the ability to retrospectively input data in text form and a reminder system. Participants also found difficulty with the units for serving sizes. While using image-based input, the editing options were in whole units, for example, ‘1 medium apple’, which could be increased in increments of 0.5. When using text-based input or editing a food item, these units defaulted to weight in grams. Participants found accurately estimating the serving size by weight challenging and reported a preference for whole units of food items where possible. Difficulty with estimating portion sizes and weights is an established obstacle in dietary assessment, with the accuracy of estimates varying by gender, age, education, portion size, and characteristics of the food item; that is, discrete food items like apples are more accurately estimated than non-discrete food items like soup.^(39,40)^ Serving size estimation can be assisted or automated using various computer vision methods, with a number of these approaches showing promise to accurately estimate serving size while reducing participant burden.^(41)^ However, even an accurate automated estimation will not aid participants when recording after the food item has been consumed. In this setting, portion size estimation aids such as images or reference objects projected in augmented reality can improve the accuracy of serving size estimation.^(40)^

### Strengths

This study had several strengths. The study population was diverse and included a high proportion of Māori participants (41%), compared to the national population of 17.8%.^(42)^ This enables future iterations of the App to be informed by Māori perspectives to increase relevance and acceptability. The inclusion of the 24-h recalls allowed the comparison to a widely used and validated dietary assessment tool. The user experience questionnaire identified a number of areas for improvement in subsequent iterations of the App.

### Limitations

The study relied on a comparison between EI and expenditure. While they are theoretically equivalent in weight-stable individuals, this balance is only observed over at least a week, and may not have reached equilibrium in some or any of the participants.^(43)^ This is of particular importance in this study, as it was recruited from a larger dietary intervention trial examining metabolic change, which, although it wasn’t energy restricted, did result in modest weight loss over 12 weeks. This is a limitation of any study examining the validity of a dietary assessment method; even the gold-standard method of validating dietary assessment methods, doubly labelled water, estimates EE.^(44)^ As mentioned above, this study compared 7 d of App use against two 24-h recalls. While this was a pragmatic decision, the fewer number of days assessed increases the effect of day-to-day variability in EI estimations for the 24-h recall. Additionally, the food composition databases differed between the App and the 24-h recall; the App uses the proprietary Passio database, while Intake24 uses an adapted New Zealand Food Composition database.^(17)^ This could result in identical reported intake producing different estimated EIs across the two methods. The participant’s experience was also negatively affected by App bugs and crashes.

### Conclusion

This study suggests that the EI estimates of free-living individuals from the App are comparable to those of the 24-h recall method, a previously validated tool that is widely used in nutritional research. Although the App was generally well liked, participants have suggested several App features and user experience changes to improve the App and will be incorporated into the next version. These modifications may further improve the accuracy of EI estimation. This study has highlighted the need for dietary assessment tools to integrate with a food composition database specific to the population of interest.

## Supporting information

Lee et al. supplementary material 1 Lee et al. supplementary material

Lee et al. supplementary material 2 Lee et al. supplementary material

Lee et al. supplementary material 3 Lee et al. supplementary material

## Supplementary material

For supplementary material accompanying this paper visit https://doi.org/10.1017/jns.2025.10045

## Appendix

Members of the He Rourou Whai Painga Consortium : Jeremy D. Krebs (University of Otago Wellington, Centre for Endocrine, Diabetes and Obesity Research (CEDOR), Wellington Regional Hospital — Te Whatu Ora); Richard Gearry (University of Otago Christchurch); Troy L. Merry (The University of Auckland, Maurice Wilkins Centre for Molecular Biodiscovery, The University of Auckland); Andrea Braakhuis, Anna Worthington (The University of Auckland); Fiona E Lithander (Liggins Institute, University of Auckland); Meika Foster (Liggins Institute, University of Auckland, Edible Research Ltd); Anna Rolleston (Manawaora Integrated Health and Research Ltd); Amber Parry-Strong, Cecilia Ross (Centre for Endocrine, Diabetes and Obesity Research (CEDOR), Wellington Regional Hospital — Te Whatu Ora); Mark Weatherall (University of Otago Wellington); Denise Conroy (Plant and Food Research); Cheryl Davies (Tū Kotahi Māori Asthma and Research Trust, Kōkiri Marae)
