# Supplementary material for: An automated image-based dietary assessment application: a pilot study
Source: J Nutr Sci. 2025 Nov 4;14:e75. doi: 10.1017/jns.2025.10045 (PMC12658290; doi:10.1017/jns.2025.10045)
Supplement: Lee et al. supplementary material 1 — Lee et al. supplementary material [file S2048679025100451sup001.docx]

**Appendix (A): Main functionalities of the application**


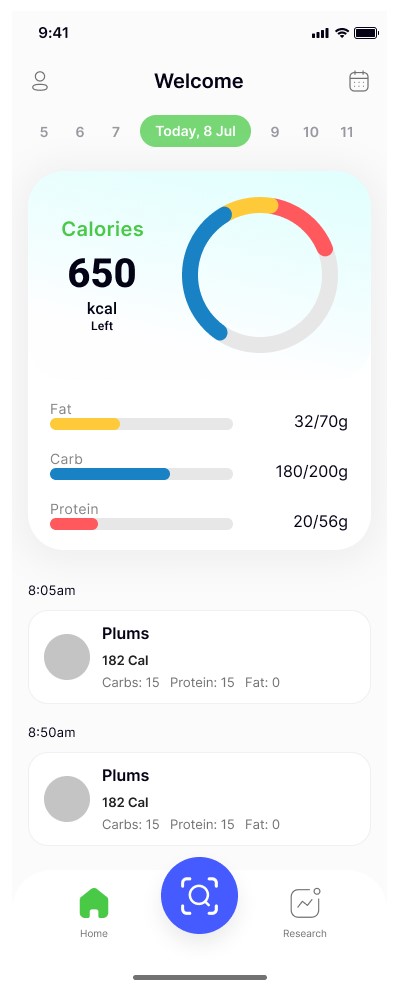

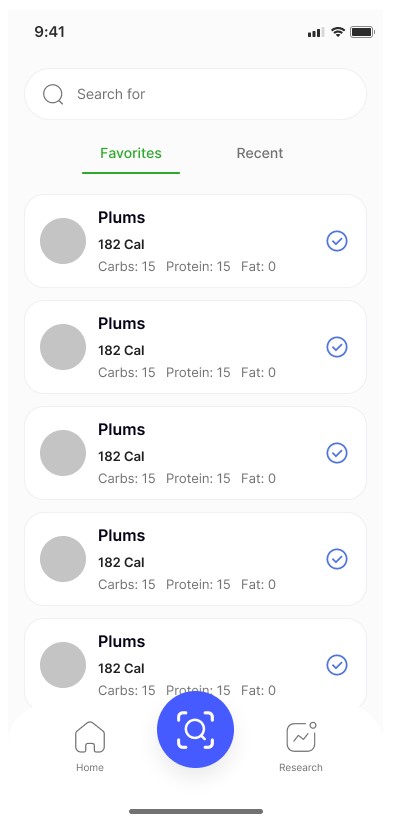

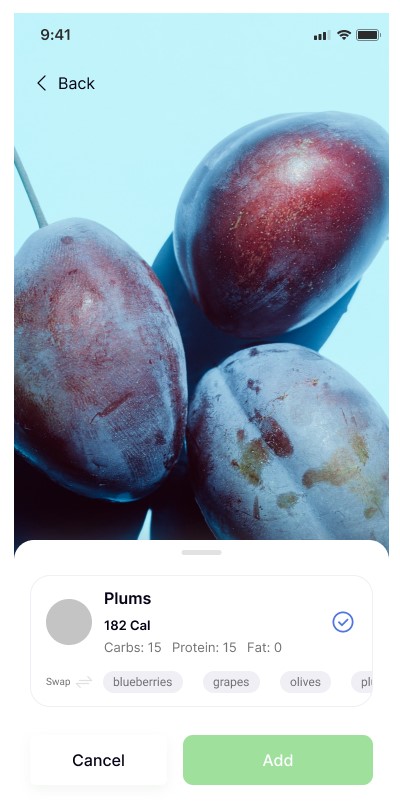


**(a)** Dashboard **(b)** Text-based input **(c)** Image-based input

**Supplementary Figure:** The three main functionalities of the application
