# Supplementary material for: An automated image-based dietary assessment application: a pilot study
Source: J Nutr Sci. 2025 Nov 4;14:e75. doi: 10.1017/jns.2025.10045 (PMC12658290; doi:10.1017/jns.2025.10045)
Supplement: Lee et al. supplementary material 2 — Lee et al. supplementary material [file S2048679025100451sup002.docx]

**Appendix B: User Experience Questionnaire**

This is a short questionnaire looking at your experience using the app. If you have any questions about the questions here don’t hesitate to ask a researcher ([leela041@student.otago.ac.nz](mailto:leela041@student.otago.ac.nz))

1. How would you described your overall experience with the app?
   1. Free text entry response
2. Have you had any experience with other food tracking apps? If so, which apps and what was your overall experience with them?
   1. Free text entry response
3. What did you like most about the app?
   1. Free text entry response
4. What did you like least about the app?
   1. Free text entry response
5. Did anything surprise you about using the app?
   1. Free text entry response
6. Did anything frustrate you about the app?
   1. Free text entry response
7. Did you think any features were missing from the app?
   1. Free text entry response
8. How likely is it that you would use the app in the future?
   1. Visual analogue scale response, from 0 (Not likely at all) to 100 (Very likely)
9. How likely is it that you would recommend the app to a friend or family member?
   1. Visual analogue scale response, from 0 (Not likely at all) to 100 (Very likely)
10. How accurate did you find the ‘Scan’ (image-based input) feature in identifying your food item?
    1. Visual analogue scale response, from 0 (Not accurate at all) to 100 (Very accurate)
11. How often could you find your food item in either Search (text-based input) or Scan (image-based input)?
    1. Visual analogue scale response, from 0 (Never) to 100 (Very often)
