# Supplementary material for: An automated image-based dietary assessment application: a pilot study
Source: J Nutr Sci. 2025 Nov 4;14:e75. doi: 10.1017/jns.2025.10045 (PMC12658290; doi:10.1017/jns.2025.10045)
Supplement: Lee et al. supplementary material 3 — Lee et al. supplementary material [file S2048679025100451sup003.docx]

**Appendix (C): Supplementary figures and tables.**

**Figure A:** Bland-Altman plot of EI_recall_^a^ and estimated EE^b^.


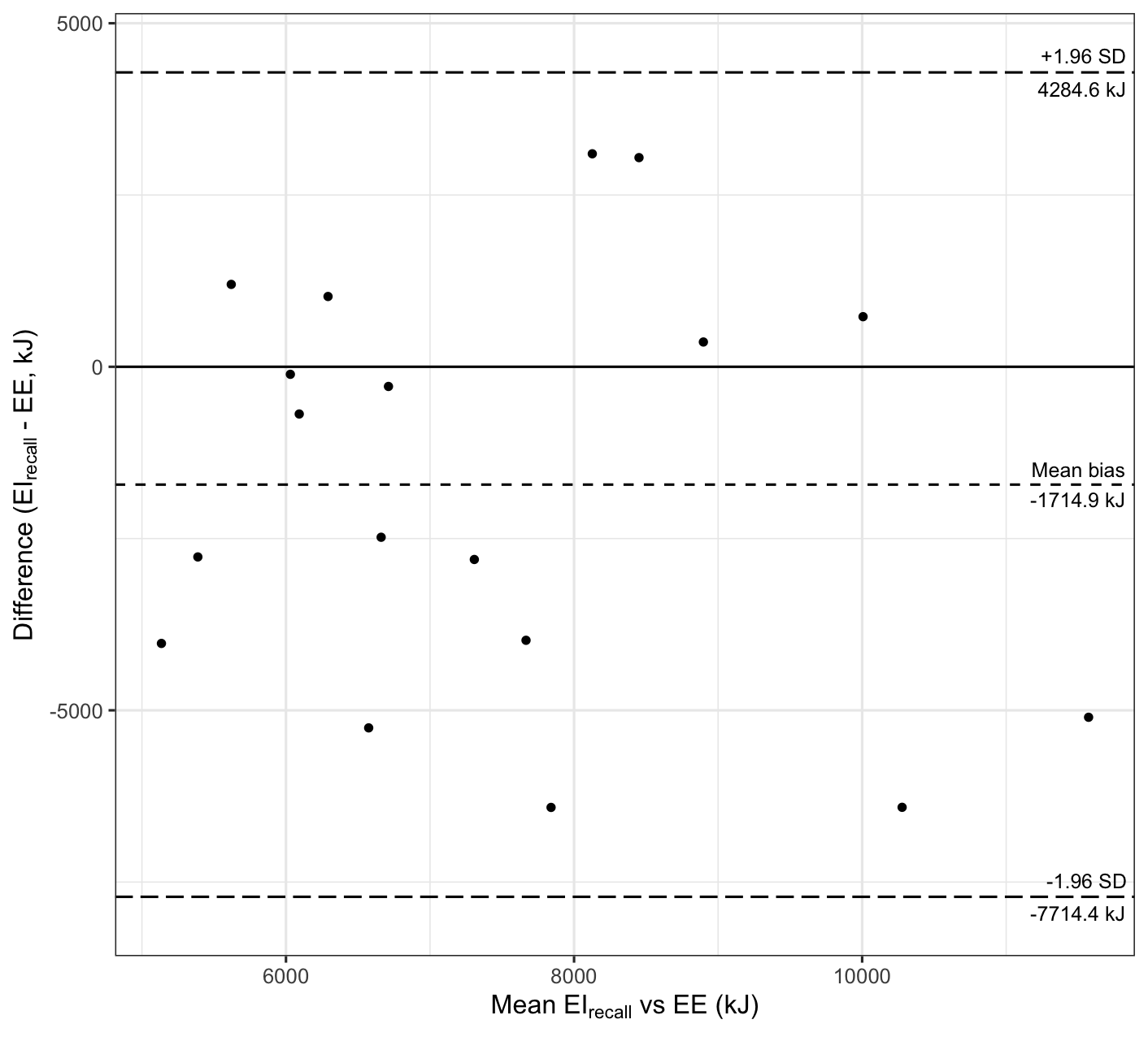


^a^ EI_recall_: mean of energy intake between two 24-hour recalls completed within 30 days

^b^ EE: mean of energy expenditure measured by indirect calorimetry and accelerometery across the observation period

**Figure B.** Bland-Altman plot of EI_app_^a^ and EI_recall_^b^.


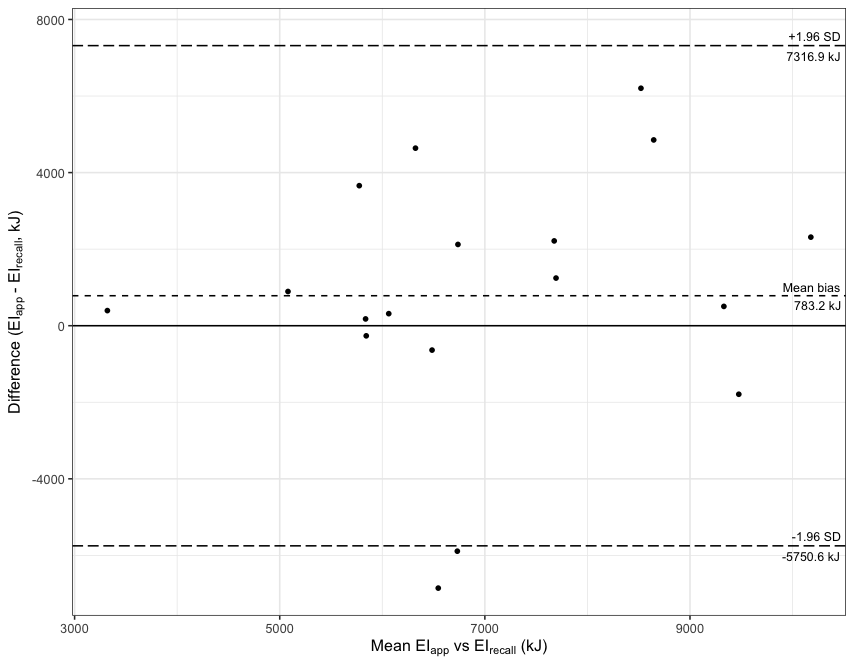


^a^ EI_app_: mean of daily energy intake across the observation period

^b^ EI_recall_: mean of energy intake from two 24-hour recalls

**Table A.** Mean and SD of EI_app_^a^ and EI_recall_^c^ in kilojoules

| **Sex** | **Mean EI_app_ (SD, n)** | **Mean EI_recall_ (SD, n)** |
| --- | --- | --- |
| Male | 8480 (1877, 5) | 7028 (1231, 5) |
| Female | 6993 (2789, 13) | 6467 (2481, 13) |
| Total | 7406 (2605, 18) | 6623 (2184, 18) |

^a^ EI_app_: mean of daily energy intake across the observation period.

^b^ EI_recall_: mean of energy intake from two 24-hour recalls.
